# “Am I a priest in the armed forces, or a soldier who is a priest?” Identity work among military chaplains in the Swedish armed forces

**DOI:** 10.3389/fsoc.2025.1645776

**Published:** 2025-10-22

**Authors:** Jan Grimell

**Affiliations:** Department of Sociology, Faculty of Social Sciences, Umeå University, Umeå, Sweden

**Keywords:** military chaplain, military chaplaincy, identity work, hybrid profession, institutional complexity, military culture, identity

## Abstract

Military chaplains (MCs) in Sweden embody a hybrid professional identity at the intersection of ecclesiastical and military institutions. Ordained Lutheran priests serve within the Swedish Armed Forces, where they may carry arms for self-defense—challenging conventional boundaries between religious and military roles. This study investigates the identity work undertaken by Swedish MCs as they navigate this complex terrain. Based on qualitative data from a 2025 study involving 50 Swedish MCs, this article adopts a narrative approach to understanding identity work in hybrid professional roles. An anonymous qualitative questionnaire enabled participants to reflect openly on their experiences of negotiating the contrasting demands of military and clerical life. The findings reveal that Swedish MCs engage in continuous identity work to maintain clerical integrity while adapting to military culture. Entering the Armed Forces necessitates the deliberate acquisition and internalization of military culture, including its values, hierarchies, practices, and norms, while simultaneously preserving the integrity of the priestly identity, perspective, and commitment. Military culture, shaped by discipline, loyalty, and the potential use of lethal force, both challenges and transforms aspects of MCs’ identities. Carrying weapons intensifies underlying conflicts and constitutes a particularly charged locus of ethical and identity-related tension, which may also generate cognitive dissonance. Furthermore, MCs often inhabit a liminal position, fully belonging neither to the military community nor to the ecclesiastical sphere, which necessitates ongoing negotiation of professional boundaries and personal values. This study contributes to the sociology of hybrid professions and institutional complexity by illustrating how implicit work contracts and organizational cultures shape identity work in boundary-spanning roles. It highlights the need for structured support and reflective practice to sustain professional integrity in such demanding contexts. Future research should adopt longitudinal designs to explore how MCs’ identity work evolves over time, especially in light of shifting geopolitical contexts and increasing demands on their hybrid role.

## Introduction

Military chaplains (from here on MCs) is a relatively new generic term for an old occupation: namely, priests or other religious representatives embedded in military units to support, care for, and provide pastoral guidance to personnel deployed to war zones and armed conflicts (Carey et al., 2016). This practice is commonly referred to as pastoral care or spiritual care (Carey and Hodgson, 2018; Graham, 2017; Pleizier and Schuhmann, 2022). In a military context, however, their role is often not limited to these functions but also includes education, ceremonial duties, advisory responsibilities, and crisis support (Grimell et al., 2025a). The work performed by MCs must therefore generally be understood within the framework of regulations that structure and legitimize their roles and functions (Bock, 1998). Thus, the military organization, its culture, and context also function as a workplace for priests (both ordained and non-ordained) serving as chaplains.

The presence and employment of MCs in the armed forces of different countries are shaped by cultural, legal, traditional, and ecclesiastical or religious frameworks (Grimell et al., 2025a). In some nations, the presence of chaplains in the military—and, by extension, the institution of military chaplaincy—is rooted in centuries of tradition and experience. Sweden constitutes one such example, where Lutheran priests from the Church of Sweden have maintained an unbroken presence in the Armed Forces for 490 years, providing support through a practice known in Sweden as *military soul care* (Grimell, 2024a).

From an international perspective, MCs are typically uniformed but generally unarmed. When required to move outside of protected military zones such as bases or camps, and when operationally feasible, they are usually accompanied by an armed soldier or provided with armed escort, based on threat assessments. The specifics of such arrangements vary across countries and operational environments.

The Nordic countries, however, represent a notable exception in this regard, as MCs may be armed as part of their chaplaincy service—even though they are ordained clergy (Liuski and Grimell, 2022). This is also the case in Sweden, where chaplains are typically armed during deployments to conflict zones, certain military exercises, or in wartime—mirroring the armament status of regular soldiers. However, they are not armed during routine duties at domestic units or garrisons. Swedish MCs hold the status of non-combatants but, through this model, can provide for their own protection and that of others when necessary (Grimell, 2020). This capability also enables them to be more present in military operations and among soldiers in order to provide *military soul care* more effectively (Grimell, 2024a). This dual role produces a *hybrid professional identity* (Watson, 2017, p. 270)—that of both priest and soldier—which warrants further in-depth investigation.

Since few Western military chaplaincy services involve armed chaplains, this has not traditionally been a subject requiring scholarly attention. However, in a Nordic—and particularly Swedish—context, the topic is increasingly relevant given the current geopolitical uncertainty, especially in light of the ongoing war in Ukraine. At present, there is virtually no research on this issue beyond Swedish studies (Grimell, 2021a).

This article aims to enhance understanding of the identity work undertaken by clergy in their hybrid professional identity as MCs, where they are required to integrate two contrasting professional identities—those of priest and soldier—and reconcile the cultural frameworks of both vocations.

The article proceeds with a description of identity work and a brief overview of military culture and identity, before addressing the potential challenges encountered by priests as they enter the military organization and its culture in their roles as MCs.

### Conceptualizing identity work

Within the sociology of work, *identity work* can be described as the process whereby individuals strive to shape a relatively coherent and distinctive sense of personal self-identity while also grappling with, and to some extent seeking to influence, the multiple social identities that pertain to them in the various contexts in which they live and work (Watson, 2017, p. 303–304).

Watson (2017, p. 304, 307-308) describes identity work as the continual processes through which people shape, negotiate, and communicate their narrative identities across varying social contexts. In this view, identity is not regarded as an inherent or permanent quality but is instead formed and re-formed through engagement with cultural norms, societal expectations, and institutional frameworks (Alvesson and Willmott, 2002). Identity, therefore, should be understood as fluid, contingent on context, and open to change as individuals move between different social settings (Sveningsson and Alvesson, 2003). Within professional life, this often entails adapting one’s narrative identities to resonate with occupational roles or organizational cultures. Such adaptation is seldom simple, as it frequently requires negotiating tensions between personal convictions and the pressures exerted by social and institutional demands (Alvesson et al., 2008). Identity work can thus be viewed as a balancing process through which individuals seek both social recognition and fidelity to their own authenticity (Brown, 2015). Ultimately, it is a dynamic—and at times fraught—endeavor in which individuals strive to construct a coherent and meaningful account of who they are (Grimell et al., 2025b).

Research on identity work often draws on people’s narratives about how they perceive themselves and how they are perceived by others (Grimell et al., 2025b; McAdams et al., 2002, 2006; Mishler, 2004; Watson, 2017, p. 307–308).

### Constructing military identity through organizational culture

Military identity is shaped through a distinct process of socialization embedded in the structure and culture of the armed forces. The concept of military identity is an analytically generic term with the capacity to encompass various types of sub-identities that emanate from military culture. Military identity functions as an overarching concept inhabited by general cultural markers of military identity (Grimell, 2024b).

Central to this identity formation is the strict hierarchical organization governed by rank, where all members are expected to conform (Hall, 2012a; Huntington, 1957, p. 16). Discipline is not merely a value but a foundational principle, and personal needs—such as rest, breaks, or discretionary time—are subordinated to the mission and the collective operational rhythm (French, 2005, p. 3–4; Hall, 2012b; Thornborrow and Brown, 2009).

Physical and psychological resilience are critical components of military identity. Service members undergo rigorous education and training designed to cultivate endurance, physical fitness, strength, and operational competence (Brotz and Wilson, 1946; Woodward and Jenkings, 2011). Achievements that demonstrate extreme toughness and capability are not only rewarded but revered, reinforcing a performance-driven ethos within the military sphere.

The collective holds primacy over the individual. From initial training through to deployment, the military fosters a moral order in which loyalty to the unit surpasses personal interest (French, 2005, p. 7; Hall, 2012a,b; Huntington, 1957, p. 8–9). This collectivist orientation becomes deeply ingrained during conscription or basic training and continues to inform behavior and self-understanding throughout a service member’s career (Grimell, 2023).

Loyalty within military culture extends beyond institutional structures to interpersonal bonds—particularly those forged in combat. The commitment to comrades-in-arms, often referred to as “battle buddies,” reflects a profound sense of solidarity and mutual responsibility. In some cases, this bond is described in terms that rival the gravity of religious vows, such as the implicit promise to remain united ‘until death do us part’ (French, 2005, p. 12; Lunde, 2009; Sørensen, 2011).

A fully formed military identity also encompasses the capacity to engage in or support lethal operations. Participation in combat—either directly or indirectly—requires service members to overcome the civilian taboo against killing, an act sanctioned and legitimized by military culture in specific operational contexts (Bragin, 2010; French, 2005, p. 4; Goldstein, 2001, 9–10, 127; Strachan, 2006; Verrips, 2006; Wilson, 2008). Within this framework, a warrior ethos may emerge that elevates mission focus, physical power, and combat effectiveness to the status of embodied virtues (Edström et al., 2009, p. 23, 30; Malmin, 2013).

### A strong priestly identity negotiating uniform and arms

In most cases, belief in God is deeply interwoven with the self; it constitutes a core identity position that influences the broader identity matrix of the believer. This belief is simultaneously personal and social—it is rooted in belonging to a congregation, aligned with a belief system, and embedded in values, meanings, and religious practices (Ideström, 2009; Hammar, 1981). It is fair to say that priests generally believe in a way that aligns with the description above. They believe to such an extent that they choose to dedicate themselves to this professionally—to preach about God, officiate weddings, perform baptisms and funerals, provide pastoral care and counseling, and so on (Ideström, 2009; Hammar, 1981).

In addition, the path to becoming an ordained priest in the Church of Sweden entails a rigorous selection and educational process. This includes 4 years of theological studies at a university level, a period of practical parish-based training as a priest candidate, and 1 year of pastoral formation at the Church of Sweden’s Educational Institute, culminating in ordination. This process generates a distinct and robust personal and social identity anchored in the ministry of being a priest.

When Swedish priests take up military chaplaincy roles within the Swedish Armed Forces, they enter a social and cultural lifeworld that stands in contrast to that of parish ministry. In this military setting, chaplains are expected to learn and adapt to military culture. They may carry weapons, receive weapons training, and undergo limited combatant education, even though they remain formally classified as non-combatants and are permitted to act only in self-protection or self-defense. This cultural contrast lays the foundation for *identity work* (Watson, 2017, p. 303–308) among ordained priests transitioning into military chaplaincy (Grimell, 2020).

Military chaplaincy in Sweden requires priests to come to terms with significant self-identity work, as they navigate a military identity that, at times, contrasts starkly with the theological and pastoral foundations of their vocation (Grimell, 2021a). The notion of an armed priest who may, under certain circumstances, be prepared to use lethal force while simultaneously performing ecclesial duties, creates a potential for conflict on multiple levels—personal, professional, social, ecclesiastical, and theological.

On a personal and professional level, the act of killing stands in fundamental tension with the ordination vows taken by priests in the Church of Sweden—vows that form the normative foundation of their ministry and identity as clergy (Grimell, 2021b). This tension is further reflected in the social sphere, where military defense and the use of lethal force are not naturally integrated into ecclesial life outside the armed forces. As a result, MCs may be perceived as anomalies among ordinary clergy and congregations. At the same time, their non-combatant status within the military can make their hybrid professional identity appear somewhat distinct or atypical within the broader military environment. As a result, they are neither typical priests nor typical soldiers, but rather occupy a position in between.

Ecclesiastically, the Swedish context is marked by the primacy of church law over secular legislation when it comes to the office of priesthood. The Church retains full authority over the regulation of priestly conduct, and a priest found to have violated ordination vows may be stripped of the right to serve by the diocesan chapter (*Domkapitel*), even if the act in question is legally permissible under civil law or unrelated to the priest’s formal employment under Swedish labor law. As a theoretical example, a MC may, according to all legal frameworks, be fully entitled to kill an enemy in self-defense as an armed non-combatant. However, if this is not compatible with the vows of ordination, then church law takes precedence over secular law (Grimell, 2024a).

Theologically, the issue is no less fraught. The use of lethal force by a priest may raise serious objections grounded in pacifist interpretations of the Christian gospel of peace. At the same time, Christian tradition also contains robust theological arguments in support of self-defense, most notably in the form of the “just war” doctrine (Holmes, 2005). Military chaplaincy thus inhabits a morally and spiritually ambiguous space, one that challenges conventional boundaries between priesthood and military service and calls for careful, context-sensitive interpretation.

## Method

The descriptive material on which this article is based derives from a study of Swedish MCs, conducted in March 2025 as part of a research project commissioned by the Swedish Armed Forces’ Chief of Chaplains. The aim of the study was to deepen the understanding and knowledge of Swedish MCs from a contemporary perspective. Given the limited qualitative research on this professional group in the present-day context, a qualitative approach was deemed particularly appropriate in relation to the study’s objective.

To build upon previous Swedish research while also gaining greater breadth and depth, a qualitative questionnaire was selected as the data collection method (Kvale and Brinkmann, 2015; Flick, 2018). This approach allowed for the inclusion of a relatively large number of participants, while also offering the kind of in-depth, open-ended responses typically associated with qualitative interviews (Kvale and Brinkmann, 2015; Flick, 2018). It provided a valuable overall insight into the experiences of MCs while also allowing participants the space to express themselves freely and in their own words. Moreover, the questionnaire was entirely anonymous, which reduced potential barriers to expressing personal experiences, thoughts, and emotions.

The study was ethically reviewed and approved by the Swedish Ethical Review Authority (Reference number: 2024–08417-01).

### Specific questions on military culture and identity

The qualitative questionnaire included a thematic section titled *Military Culture and Identity*, comprising three open-ended questions explicitly designed to explore participants’ experiences and reflections on their identities as clergy and their roles as MCs within a military context. The questions were:

- In what ways do you feel that military culture (values, meanings, practices, uniform, etc.) has influenced you from an identity perspective?
- Do conflicts or tensions ever arise between your ecclesiastical and military roles? (If yes, please describe)
- How do you perceive the compatibility between theology and the military profession, including operations and warfare?

These three questions form the analytical foundation for the findings presented in this article.

#### Inclusion criteria

Since the study was commissioned by the Swedish Armed Forces’ Chief of Chaplains, collaboration was established to ensure that the researcher could reach as many participants as possible at the same time, thereby optimizing the efficiency of data collection. The inclusion criteria for participation were as follows:

(1) Serving as a MC at one of the Swedish military organizational units,
(2) Being present at the Swedish Armed Forces Headquarters during the identified training days (i.e., not, retrospectively),
(3) Voluntarily consenting to participate in the study.

#### Implementation

The qualitative questionnaire was administered during the Swedish MCs’ training days, held on March 12–13, 2025, at the Swedish Armed Forces Headquarters in Stockholm. Since the use of electronic devices (computers, mobile phones, etc.) was not permitted in the facilities, the questionnaire was distributed in paper format. The study was introduced orally, after which each participant received written information and an informed consent form. Participation was entirely voluntary.

Participants were given 1 h and 40 min to complete the questionnaire—equivalent to the duration of a relatively long research interview. The questionnaire was entirely anonymous, and the consent forms were submitted separately to ensure that no data could be linked back to individual responses.

### Military chaplains in a Swedish context

In the Swedish context, MCs exist in several different positions. A unifying feature is that, with few exceptions, they are ordained Lutheran priests within the Church of Sweden.

One category consists of priests formally employed by the Church of Sweden who, as part of their regular ecclesiastical duties, also serve as MCs on regiments, air bases, naval stations, and garrisons within their local pastoral jurisdictions. These chaplains typically serve between 20% and 75% of their time in military settings, occasionally more, with the remaining time dedicated to parish work. For the sake of simplicity, these participants are hereafter referred to as *Regiments, Air Bases, Naval Stations, and Garrisons MC*.

Another category includes chaplains assigned to Home Guard battalions. These battalions are composed of part-time military personnel who maintain civilian careers but are highly mobilizable. MCs in this category work full-time as parish priests and additionally serve approximately 8–10 compulsory duty days per year in their assigned battalions. They therefore generally do not perform their duty days as part of their employment as parish priests in the Church of Sweden, but are instead employed by the Home Guard Battalion, i.e., the Swedish Armed Forces. These participants are hereafter referred to as *Home Guard Battalion MC.*

A third category operates at a regional command level, where a significant portion of their work is conducted. The regional MCs are attached to one of the five military regions in Sweden: North, Central, West, South, and Gotland. Each military region has a staff that leads operations during national crises and provides support to society. The employer is the Swedish Armed Forces.

There are also exceptions and extensions to these primary categories (see Grimell, 2020 for further discussion).

In addition, continuing education days are commissioned by the Chief of Chaplains for MCs of all categories.

A Swedish MC is generally required to have served for several years as a priest before becoming eligible for the assignment. They often have qualified experience in soul care and counseling. Some have a military background, either through conscription or as officers in some capacity. Certain military training is provided as part of the military chaplaincy assignment (for more information, see Grimell, 2020, 2024a).

### Overview of the participants

Table 1 below presents a summary of the participants at the group level.

**Table 1:** Participant overview.

Category	Number	Comment
Total participants	50	Represents just under half of the growing Swedish MC population
Gender	32 men, 18 women	
Age	Majority 35–55 years	A few under 35 and over 55
Current positions		
Regiments, Air Bases, Naval Stations, Garrisons MCs	17	
Home Guard Battalions MCs	24	
Sensitive MC positions (unspecified)	3	Not disclosed for ethical reasons
Did not disclose	6	Due to anonymity concerns
Additional information		
Multiple MC roles during career	21	Served in several different functions
International deployments	11	Served in conflict zones
Length of service	0–30 years	From newly appointed to very experienced
Average length of service	8.75 years	Most participants were highly experienced

To protect participant anonymity and prevent potential back-tracing, neither gender nor length of service will be explicitly reported alongside the interview excerpts, as combining such information may inadvertently disclose individual identities. This also applies to specifying which regiments, air bases, naval stations, garrisons, and Home Guard battalions the participants belong to. The same principle applies to any other potentially identifying or sensitive information (e.g., specific deployments, ranks, duty stations, or particular events), which has been omitted or anonymized where necessary. This was particularly pertinent given that participants were assembled at the very same occasion when completing the qualitative questionnaire, although they were given the option to leave the room and complete it in a different setting.

### A narrative concept of identity

This article adopts a narrative approach to analyze how participants construct and express identity claims through their identity work. Both narrative psychology (McAdams et al., 2002, 2006), sociology (Grimell, 2024b), and work sociology (Watson, 2017, p. 306–307) emphasize how identities are constructed through storytelling processes and narrated claims about who individuals are. Individuals make sense of their working lives by narrating stories about themselves—so-called *self-narratives*. These narratives help individuals understand who they are, what their work means, and how they relate to others and to broader institutional contexts (Watson, 2017, p. 306–309).

Identity, from a narrative perspective, is understood as negotiated and contextual, dynamic and continuously shaped through the interaction between individual agency, organizational structures, and societal frameworks (Watson, 2017, p. 307).

Identity work is conceptualized as the process whereby participants strive to shape a relatively coherent and distinctive sense of personal self-identity as both clergy and armed within a military culture where self-defense as a non-combatant is part of the militarized chaplaincy identity. At the same time, they attempt to come to terms with—and, within limits, to influence—the various social identities attributed to them within the ecclesiastical and military milieus in which they live and work.

In the case of MCs, it remains both a methodological and analytical question whether they are navigating two distinct identities—one as a priest and one as a military but non-combatant figure—or whether they are best understood as integrating military elements into a pre-existing clerical identity, thereby forming what may be termed a hybrid professional identity (Watson, 2017, p. 270). Both interpretations are supported by the material in this study. Regardless of whether one argues for the presence of two distinct identities or a hybrid MC identity, the identity work undertaken by MCs involves a continuous *negotiation* and *dialogue* (Grimell, 2024b; Hermans, 1996, 2001, 2002) between a salient priestly identity and a position within, or elements drawn from, a military culture—one that encompasses specific military components such as values, meanings, practices, uniform, and weapons.

### Researcher background and reflexivity

The researcher, a senior lecturer, has a background both as a tactical officer (approximately 14 years) and as a priest (approximately 6 years) in the Church of Sweden. This dual experience has provided valuable conditions for understanding and relating to the respective organizations, cultures, and professional identities of the two institutions. Over the past decade, the researcher’s scholarly work has focused partly on identity processes, reconstruction, and negotiation during the transition from military to civilian life among military personnel, and partly on military chaplaincy. In the present study, however, the identity process takes the opposite direction, namely entering the military context, where an established clerical identity must coexist alongside a military one. Previous research on identity processes has proven particularly useful in making sense of the identity work that MCs engage in during this process, which in turn mirrors, though in reverse, the experiences of active military personnel transitioning into civilian life.

The researcher’s previous decade of qualitative research and analytical activity, together with the use of theoretical triangulation (Ramakrishnan et al., 2023), whereby the qualitative findings is analyzed through multiple theoretical perspectives such as identity work, identity concepts, and work-sociological notions including the implicit work contract, hybridity, complexity, and the boundary spanning profession, has contributed to rigor by illuminating the findings from multiple vantage points.

### Coding of qualitative data

The initial step in the coding process involved transferring all handwritten responses from the paper-based questionnaire into a digital Word format. This was necessary in order to process the entire dataset using the qualitative data analysis software Atlas.ti.

Although only one thematic section comprising three questions is addressed in this article, the full questionnaire consisted of 31 questions spread across nine pages. In cases where the space provided was insufficient, participants were encouraged to write on the reverse side of the pages. As a result, the material amounted to approximately 450 pages of responses, along with numerous back pages, all of which required transcription into Word documents.

Responses were transferred on a per-question basis, meaning that all responses to a single question were compiled into one document. Each response was tagged with an individual participant code to maintain traceability. This was a time-consuming but essential preparatory task.

The next phase involved importing the individual Word documents—each containing responses to a specific question—into Atlas.ti for coding and analysis. A thematic analysis with an inductive approach (Elo and Kyngäs, 2008; Grimell, 2020, 2024b; Thomas, 2006) was conducted using the Atlas.ti software. This process consisted of two main steps: initial inductive coding, followed by a more structured thematic organization of the codes.

Initial coding entailed identifying and labeling specific observations in the responses, guided by the three focal questions: (1) how military culture affected participants from an identity perspective; (2) experiences of conflict between ecclesiastical and military roles; and (3) how participants reconciled theology with military practice. Coding was thus performed in close proximity to the empirical material. For example, a statement such as “there is a clear hierarchy” was coded as *clear hierarchy (military)*, and a remark like “I carry two identities. One in clerical collar, one in uniform.” was coded as *one priest and one military identity*, and so forth.

These individual codes were subsequently aggregated into broader, more abstract *code families*—as termed in Atlas.ti—in order to enable a meaningful thematic analysis focused on central aspects of MCs’ identity work within the military context. While moving from the micro-level of coding to higher-order thematic categories inevitably entailed a loss of some of the nuanced detail, this step was necessary to facilitate a coherent and analytically meaningful presentation of the findings.

Table 2 below presents the five code families, which also serve as categories or themes that organize the subcodes.

**Table 2:** Primary categories underpinning identity work.

Primary category/theme	Subcategories
Point of entry	Military vs. civilian background
Experiencing military culture	No salient subcategories, only codes within the primary category
Becoming “one of the group” through learning military cultural performance	No salient subcategories, only codes within the primary category
Balancing closeness and distance (as a means of upholding integrity)	No salient subcategories, only codes within the primary category
Conflicts and tensions between the ecclesiastical and military aspects of identity	• Using weapons as an MC • Working hours and employer relations • Like an island: neither fully military nor fully a parish priest • Cultural jargon • Gender • Other social experiences

The analysis presented below will use these categories as headings to unfold the material and weave it together, all the way back to the empirical material through excerpts from the coding of the qualitative questionnaire.

## Findings on identity work among participants

**Figure 1:**
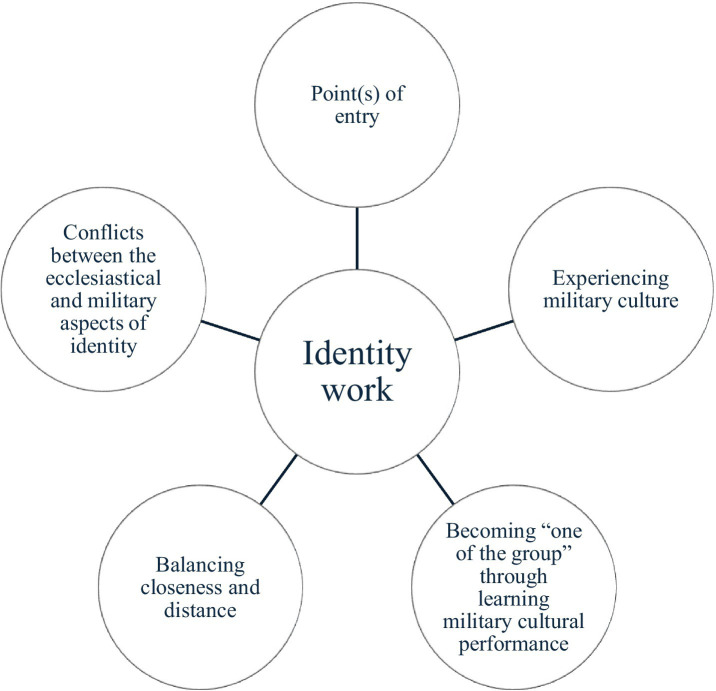
Categories that underpinned the identity work.

The five overarching analytical categories or themes formed the basis for the identity work in which the participants needed to engage, in accordance with Figure 1.

Entering the military lifeworld as a priest from the Church of Sweden entailed encountering a culture of strong contrasts and undergoing a socialization process in which the priest gradually learned to understand military values, meanings, and practices. However, given their role, priests could not adopt a neutral and external observer position; rather, they needed to become part of the organization, culture, and military community to effectively fulfill their mission. This meant learning to wear the uniform, mastering military terminology and practices, and adopting a military mindset. At the same time, it was crucial for the priests to maintain their identity and integrity as clergy—thus, balancing both closeness and distance was essential.

### Entry points to experiencing military culture

The integration process varied among participants, as they entered their assignments as military chaplains with diverse backgrounds. Several had previously completed conscription or had a more prominent military background, for instance through deployments to conflict zones in military positions or as officers. For these participants, identity work involved a return to an already familiar military culture and a previously established military identity. Re-entering a military cultural context often reactivated this earlier identity as soldier or officer. Consequently, participants needed to negotiate and recalibrate the balance between their military and priestly identities.

Participant 3 (Home Guard Battalion MC) recounted:

I am a [military rank] and, in the first few years, I had quite a strong soldier identity. I was probably a bit too ‘cool’ at first. Soldiers handle combat. I now realize that I have different duties. I have matured significantly and now focus more on being a priest. But I am glad and proud that I can act as a soldier and handle equipment correctly.

Participant 15 (Home Guard Battalion MC) testified:

I have prior military experience. Conscription, multiple deployments to [names of mission areas and units]. I have always felt comfortable in military culture, and my earlier experiences have shaped me a great deal.

For these participants, their military identity had preceded their priestly formation and theological education, which occurred later in life. In such cases, it is analytically useful to conceptualize these as two distinct identities coexisting side by side. Identity work as a MC, for these individuals, involved creating a dialogue between these identities and finding a constructive balance where the priestly identity—not the soldier identity—would predominate in military contexts.

For priests without a military background, socialization into military culture required a more fundamental learning process. These participants became MCs after their theological training, with no prior military experience (e.g., conscription, officer training, or deployments). They thus approached military culture from a different point of entrance, without having established a distinct military identity beforehand.

The encounter with military culture, and thus the salient markers of a military identity, was described by the participants in the following ways:

Participant 8 (Home Guard Battalion MC) stated:

Clarity and defined structures!

Participant 22 (Home Guard Battalion MC) recounted:

Military culture is usually about ‘order, structure, and discipline,’ and decisions that are made are followed.

Participant 14 (Home Guard Battalion MC) stated:

Duty, dedication, order and discipline, straightforwardness, and task orientation.

Participant 45 (preferred not to state their position) testified:

It is very important that attire is correct. You must not mix clerical clothing with the uniform.

Participant 25 (Regiments, Air Bases, Naval Stations, and Garrisons MC) stated:

The Armed Forces—in both small and large ways—always have a purpose in their culture/values, and this often directly or indirectly relates to saving lives in wartime. Even a small glove can make a big difference.

Participant 39 (Regiments, Air Bases, Naval Stations, and Garrisons MC) had observed elements of the culture with a clear downside:

There is a clear hierarchy, which I sometimes find difficult, and a lot of ‘right and wrong’ that is not always important. There is also a distinct macho culture that I struggle with, and a romanticization of alcohol that I strongly oppose. At parties, military personnel can get completely drunk and behave however they like, and this is seen as entirely acceptable.

These observations resonate with recurring descriptions in the research literature of generic elements of military culture: structure, hierarchy, higher purpose, clarity, defined frameworks, duty, dedication, order and discipline, straightforwardness, task orientation, proper attire, macho culture (or warrior/strength culture) (Hall, 2012a,b, Edström et al., 2009, p. 27; French, 2005; Grimell, 2024b; Huntington, 1957, p 73–74; Lunde, 2009; Malmin, 2013; Sørensen, 2011; Thornborrow and Brown, 2009; Wilson, 2008) and the role of alcohol (Dolan and Ender, 2008; Goldstein, 2001, p. 257; Riolli and Savicki, 2010).

Entering such a culture without prior knowledge required a time-intensive process of learning and acculturation in the professional identity of MC.

Participant 5 (Home Guard Battalion MC) stated:

I am still learning military culture—I am relatively new to this role, with [several years] of experience.

Without prior knowledge, it took time to learn and navigate military culture, especially for priests serving as MCs in Home Guard Battalions, where they often had only 8–10 mandatory service days per year. To compensate, some worked voluntarily in their spare time to familiarize themselves with the organization, culture, and activities.

This was further substantiated by the fact that a core element of the ‘DNA’ of military culture lies in its social and relational bonds among military personnel, which foster a strong sense of cohesion. However, this cohesion is not readily accessible to outsiders. MCs must maintain a consistent presence in order to cultivate trust and gain acceptance among military personnel, thereby enabling them to perform their duties effectively.

### Becoming “one of the group” through cultural performance

A significant part of the identity work, regardless of point of entrance, was social and relational: entering from the civilian world and striving to become part of a tight-knit military community. The relationship between the military and the civilian world has always been complex, largely due to profound cultural differences (Bragin, 2010; Huntington, 1957, p. 11; French, 2005, p. 4; Goldstein, 2001, p. 9–10). Moreover, military communities are typically built on strong cohesion, camaraderie, collegiality, and loyalty (French, 2005, p. 12; Grimell, 2024b; Sørensen, 2011). Thus, it was no simple task for civilians to gain trust and become accepted in a military context—or, as many participants put it, to become “one of the group.”

Participant 7 (Home Guard Battalion MC) captured this sentiment:

A major advantage of wearing the uniform is that I am one of the group. We all have the same gear and the same equipment in our lockers. At the same time, it feels strange not to celebrate services in liturgical garments, etc. Am I a priest in the Armed Forces, or a soldier who is a priest?

Participant 7 also articulated a central identity question in their ongoing identity work: *Who am I in this employment and in this cultural context?* From an external perspective, one could argue that the answer is both: a priest in the Armed Forces, but also, in some sense, a soldier—both wearing the uniform and equipped as such. Identity and role questions can become pressing in professional contexts that combine elements from highly divergent organizations and contrasting workplace cultures.

One cultural attribute that was crucial for becoming “one of the group” was the uniform. The uniform embodied much of military culture: it was the shared attire of all personnel in the work environment and symbolized a concrete, rule-governed practice concerning appearance, conduct, and behavior. How the uniform was worn also conveyed something about the wearer: an experienced soldier might tilt their field cap or beret in a particular way, and mixing military and civilian garments was prohibited. The uniform also served as a marker of military identity, signaling a wealth of information (e.g., rank, unit, and specialty). More broadly, it symbolized a commitment to the defense of democracy, freedom, and liberal values in Sweden and NATO.

For identity work, the uniform also served the function of activating and deactivating a military identity:

Participant 16 (Home Guard Battalion MC) explained:

The uniform provides a sense of belonging. Being dressed only in civilian clothes marks you as an outsider—I once joined a unit while in civilian clothes. I think I take on the military identity with the uniform, and with that comes everything else.

Participant 21 (Home Guard Battalion MC) testified:

I find it quite easy to ‘step in and out,’ meaning that after an exercise, I do not walk around in uniform or speak in abbreviations.

Thus, the uniform could serve as a practical way to ‘put on’ and ‘take off’ a military identity—enabling chaplains to navigate both ecclesiastical and military contexts. Although this generally functioned well, there were notable instances in which military and ecclesial cultures, embodied through their respective identity constructs, came into tension in various ways. Deactivating military identity markers within a church context, for example, was not always a straightforward process.

Participant 30 (Regiments, Air Bases, Naval Stations, and Garrisons MC) testified:

I carry two identities. One in clerical collar, one in uniform. These often coexist without issue. But sometimes there is frustration. Sitting in a church meeting versus in the Armed Forces can be very different. For example, I may come across as harsh in church, while the opposite is true in the Armed Forces. They are different cultures. Moving between them and shifting identities takes a lot of energy.

Participant 30, who explicitly conceptualized the clerical and military as two distinct identities, illustrated how the same behavior might be perceived very differently in church and military contexts. Moreover, this was described as a demanding process, requiring the ongoing negotiation and modulation of one’s identities in response to the cultural context in which one was operating. Other participants also described conflicts when bringing military directness into the church or when wearing military attire in church settings—which could cause discomfort among congregants (e.g., refugees or immigrants with traumatic military experiences) or due to church members’ concerns about involvement with the military.

### Balancing closeness and distance

Given the influence of military culture on priests, identity work highlighted the need to engage with the culture and community with both closeness and a degree of distance. Full internalization of military culture was neither possible nor desirable; maintaining the priestly perspective, competence, and integrity was essential to contribute effectively to the Armed Forces—the ultimate purpose of their presence. If chaplains became too militarized, this would compromise their unique competencies in areas such as ethics and moral guidance. It was therefore vital to preserve intellectual and moral independence to enhance resilience and ethical robustness within the military.

Participant 35 (Regiments, Air Bases, Naval Stations, and Garrisons MC) testified:

It is important for me to always hold onto my identity as a priest. I am often ‘one of the group,’ but always a priest. I also feel that, as a priest, I can speak freely.

Participant 38 (Regiments, Air Bases, Naval Stations, and Garrisons MC) explained:

It is important to know who I am. That I am a priest. Not to become a ‘MÖP’ [a Swedish term for someone excessively interested in military matters, embodying a strongly militarized identity].

Participant 40 (Regiments, Air Bases, Naval Stations, and Garrisons MC) recounted:

I am influenced by [military culture] and generally find it positive. But, to be a priest providing military soul care where ethics matter, I believe it is also important to question certain aspects.

Participant 46 (preferred not to state their position) stated:

Military culture has influenced me to become part of it. But I have retained my core identity as a priest and have actually strengthened my sense of what I can contribute from that position.

MCs can serve as important counterparts to military culture and practice—supporting personnel from existential and spiritual perspectives, helping to uphold the laws of war, preserving human dignity in conflict, advocating for humanity in war, and advising commanders on ethical decision-making. To do this, chaplains must maintain their own moral compass and integrity, even as they navigate ongoing cultural socialization within the military. This necessitates deliberate and sustained effort, as chaplains themselves are not immune to the potential dynamics of radicalization that may be embedded within military culture.

### Conflicts or tensions between the ecclesiastical and the military

Identity work that was drawn toward conflicts or tensions typically stemmed from personal, hands-on experiences within military contexts. It did not generally concern large, overarching questions—such as whether theology can be reconciled with military practice. In fact, participants overwhelmingly considered it compatible to defend and protect oneself against an opponent—whether justified theologically (e.g., through the notion of a ‘just war’ (Holmes, 2005), through pastoral Lutheran theological reasoning (e.g., ‘the Church is where the people are’), historically (e.g., clergy have been part of the Swedish Armed Forces since the 16th century (Grimell, 2024a), or in other ways [e.g., preserving and defending human dignity in war (Grimell et al., 2025)]. This compatibility was not regarded as unproblematic or simple—but it was nevertheless seen as possible and acceptable. A few exceptions to this view were noted.

#### Using weapons as a MC

At a personal level, however, conflicts did arise concerning military culture and the militarization of MCs—specifically around the issue of carrying weapons and, by extension, the potential use of lethal force. MCs hold a non-combatant status but are armed for self-protection (though not in the course of day-to-day work).

For several participants, this aspect of identity work involved both theological and personal reflections on the act of potentially killing in self-defense and on how carrying a weapon shaped their role as MC in the field. Some of these tensions are articulated below:

Participant 5 (Home Guard Battalion MC) explained:

Of course, it feels difficult to neutralize an enemy—to be prepared to kill—even though for us it is about self-defense, defensive action. That can create a moral conflict.

Participant 20 (Home Guard Battalion MC) testified:

I think the crosses on my uniform remind me of why I am in the military. But the pistol is a difficulty. What kind of priest am I if I shoot someone so that I can survive? What kind of priest am I if I do not shoot and do not make it home? It’s tricky.

Participant 31 (Regiments, Air Bases, Naval Stations, and Garrisons MC) recounted:

Yes [in response to whether this can create conflict]. I am armed in the field and can handle my weapon. I am very careful to often remind myself and others that I am a non-combatant—often, I think I do this for myself. It is easy to become too warrior-like in trying to fit into the unit if I am not careful about my role and task as a priest in the military.

It is worth noting that Participant 31 routinely employed the mantra ‘I am a non-combatant’ as a deliberate strategy of identity work, aimed at keeping the aspects of military identity tied to weapon use in check and preventing them from becoming overly dominant. This was particularly salient in a military context where cohesion is exceptionally strong, fostering a high degree of conformity to cultural and group norms (Grimell, 2021b). Within this environment, the MC must continually negotiate a careful balance between adaptation and relational closeness, on the one hand, and professional distance and personal integrity, on the other.

For the sake of clarity, it should be noted that MCs serving at Regiments, Air Bases, Naval Stations, and Garrisons are generally not issued a personal weapon, unlike Home Guard Battalion MCs or those deployed to conflict zones. However, nothing prevents them from using or firing weapons in their role as an MC, such as during shooting exercises.

Tensions could also arise from comments or reactions from military personnel, especially when they lacked knowledge about the chaplains’ non-combatant status. Occasionally, questions would be raised about why a priest was armed.

Most participants had arrived at a pragmatic stance: this was part of the Swedish model of military chaplaincy. They employed framing strategies (e.g., viewing it as self-protection, defensive only) and gradually integrated this aspect into a hybrid professional identity as MC. While this did not entirely resolve the underlying tension, it significantly reduced cognitive dissonance. A number of participants did not address the issue of weapons at all.

#### Working hours and employer relations

Another hands-on issue that led to conflicts concerned restrictions on working hours and challenges related to employer arrangements. This also needs to be understood in context: the Swedish Armed Forces, given the current geopolitical uncertainty and the war in Ukraine, is undergoing a period of rapid expansion. New units are being established, and conscription is accelerating, resulting in a significantly increased need and demand for MCs.

The Swedish military chaplaincy model is relatively complex. In the category of *Regiments, Air Bases, Naval Stations, and Garrisons MCs*, for example, chaplains are generally employed by the Church of Sweden. In their role as parish priests, their work as MCs typically constitutes between 20% and 75% of their total employment. Depending on operational needs and demand, this was sometimes perceived as insufficient. Nonetheless, this category typically spends considerably more time on base than the *Home Guard Battalion MCs*, who are formally employed by the Swedish Armed Forces but serve as MCs for only about 8–10 mandatory days per year, in addition to their full-time roles as parish priests. This was often experienced as too little time and a source of frustration, as participants wished to perform their chaplaincy duties as effectively as possible but felt constrained by these limitations.

Participant 2 (Home Guard Battalion MC):

Yes, there is definitely a conflict—there is strong reluctance to grant time for military duties.

Participant 26 (Regiments, Air Bases, Naval Stations, and Garrisons MC) recounted:

The main conflict is about time, and there can be tensions between the two employers concerning time allocation. It is stressful.

Participant 32 (Regiments, Air Bases, Naval Stations, and Garrisons MC) stated:

No conflicts other than those related to time.

These kinds of interest conflicts generate tensions and loyalty dilemmas, which form part of the identity work and the negotiation between ecclesial and military roles—a process that participants described as stressful and frustrating. The time that a parish employer grants for a *Regiments, Air Bases, Naval Stations, and Garrisons MC* is subtracted from their regular parish duties. The parish employer may have limited motivation to reduce parish work in favor of military service. For *Home Guard Battalion MCs*, it is not entirely straightforward for the Armed Forces to alter the number of mandatory duty days. Moreover, this category of chaplains also maintains a full-time position as parish priests in the Church of Sweden.

Finally, it should be noted that priests of the Church of Sweden cannot be employed by another employer to perform priestly duties, as only the Church of Sweden holds the right to confer and oversee the exercise of the priesthood. Consequently, the Armed Forces cannot employ Church of Sweden priests in full-time positions that involve the exercise of the priesthood. A more viable path, however, might be to develop various models of compensation to the Church of Sweden to enable greater participation of priests serving as MCs within the Armed Forces.

#### Like an island: neither fully military nor fully a parish priest

Several participants also described experiences of a certain isolation in their role as MCs. This was partly linked to feelings of insufficient knowledge for the role and the fact that some participants were not formally employed by the Armed Forces but remained employees of the Church of Sweden.

Participant 40 (Regiments, Air Bases, Naval Stations, and Garrisons MC) testified:

At times, I feel somewhat outside the military world. I do not feel that I know enough. I wish I knew more. Being employed by the Church and not by the Armed Forces also makes things difficult at times. I sometimes feel that I do not fully belong.

At the same time, some participants expressed that despite adopting elements of military culture, uniform, equipment, and weapon handling, they never truly became full members of the military group.

Participant 33 (Regiments, Air Bases, Naval Stations, and Garrisons MC), on the negative aspects of serving as a MC:

On the other hand, the negative aspect is that I will never, truly, be part of the group.

Participant 39 (Regiments, Air Bases, Naval Stations, and Garrisons MC) expressed a sense of not belonging fully to the military world:

I feel a strong sense of belonging and loyalty in terms of my work, duty, and participation with the military. Yet at the same time, I do not feel that I am one of them or fully part of that world.

Participant 9 (Home Guard Battalion MC), by contrast, expressed a sense of isolation within the parish context:

In my parish work, I sometimes feel like an isolated island and a bit alone.

Such experiences of identity and role conflicts and confusion illustrate the hybrid nature of professional identity more broadly (Watson, 2017, p. 269–270), and of military chaplaincy in particular. As priests, chaplains never truly become full members of the military group. This statement, of course, warrants further nuance—what does it mean to be *fully* part of the group? If one assumes that combatant status—i.e., the capacity and legal right to engage in combat—defines what is *authentically* military, then the chaplain will always remain *in between*: neither fully civilian nor fully combatant. At the same time, however, this issue has more nuances than that. It is entirely possible to be a combatant and still not be accepted or feel like one of the group. In a male-dominated military world, gender or other perceived deviations from the cultural norm (such as deteriorating mental health or perceived weakness) can unfortunately also have this kind of social effect (Goldstein, 2001; Grimell, 2021b, 2024b).

At the same time, the church-military relationship and military identity markers may be perceived as alien and incongruous in an ecclesial or parish context, where they may clash with church culture and parish life. When such perceptions became explicit, they could also generate conflict.

Participant 6 (Home Guard Battalion MC) stated:

Yes (in response to the question of whether conflicts existed). There are those who feel that the Church and priests should have nothing to do with the military.

Thus, even in this respect, the MC often occupies a space in between that of a conventional parish priest and that of a soldier. This liminal condition triggered identity work among several participants—more or less pronounced—which remained difficult to resolve.

#### Cultural jargon, gender, and other social experiences

Other sources of tension included language, jargon, and humor within military culture, which some participants found inappropriate or unsettling.

Participant 17 (Home Guard Battalion MC) testified:

At times, certain language bothers me—for example, when we refer to our own soldiers as ‘meat in boots’.

Such language tends to dehumanize soldiers, rendering them an impersonal and ultimately expendable group. An instrumental, emotionally detached language practice is common in military contexts—for instance, terms such as *neutralise the enemy* (Goldstein, 2001, p. 221, 252; Strachan, 2006; Verrips, 2006). Yet when such language was applied to one’s own soldiers, it starkly contradicted the imperative to preserve human dignity and worth.

On a personal level, there were participants who found it more difficult to embrace certain aspects of military culture, such as obedience and discipline. This was rooted in a priestly identity and mindset that gravitated toward a contrasting ecclesial culture.

Participant 19 (Home Guard Battalion MC) stated:

I struggle a bit with the military’s discipline and order, having to obey superiors and so on. I feel the church is not like that—we listen to everyone, and no one has to suffer in the same way, at least not in my church context.

Participant 19’s choice of the word *suffering*, a direct translation of the Swedish term, may be interpreted as an expression of how this participant experienced this aspect of military culture. Participants in the study also reported, as illustrated by examples in the analysis, that elements such as directness, order, and clarity did not always align well with an ecclesial context and could be perceived as harshness.

In qualitative research, nuances and individual experiences play a significant role. This is especially relevant given that armed forces tend to be male-dominated and shaped by a masculine warrior ideal (Goldstein, 2001). Some military units remain almost exclusively male. In such contexts, a uniformed woman may be objectified, sexualised, or exoticized (Goldstein, 2001). For female MCs, this can present particular challenges, as competence and expertise may be subordinated to gender identity. This adds an additional dimension to the identity work required in the role.

Other individually shaped experiences could also generate friction in identity work—for example, mistrust from psychologists or HR personnel who did not understand the role of the Church within a secular state agency, or from stressed commanders who perceived an inflation in *korum* (the military religious service typically held within the Armed Forces).

## Discussion

There are several takeaways from this study concerning Swedish MCs’ identity work that warrant further in-depth discussion.

### The significance of continuously addressing identity work

The Swedish model is characterized by a high degree of integration of MCs into military culture, particularly for those MCs who are issued a personal weapon—an arrangement that symbolically confers a degree of soldier status and an expectation of self-protection. This structural integration allows MCs to operate alongside military personnel and to become closely embedded within military units, thereby fostering both a profound understanding of military practice and an enhanced cultural competence in delivering military soul care in proximity to military personnel (Grimell, 2020, 2024a).

At the same time, this integration entails a form of *identity work* (Watson, 2017, p. 304) that unarmed MCs in other chaplaincy services may not encounter to the same extent, although their professional identities and practice are likewise shaped by a military cultural context (Grimell et al., 2025; Carey et al., 2016; Pleizier and Schuhmann, 2022). For Swedish MCs, various forms of support for this ongoing identity work may therefore be of considerable importance, offering guidance throughout a complex and dynamic process that may also generate a form of *cognitive dissonance* between cultural identities that are not fully compatible (Festinger, 1957). Structured educational sessions addressing identity work, as well as regular and ongoing group- and individual-level dialogues on identity negotiation, appear particularly valuable—especially in view of the fact that relevant boundaries may shift as military culture itself evolves, sometimes in more radical directions (Edström et al., 2009, p. 29; Grimell, 2021b; Malmin, 2013).

Such evolving processes may not be readily perceptible to individuals embedded within units characterized by a strong and cohesive military culture and an ongoing operational tempo (Edström et al., 2009, p. 27, 30, 34; Malmin, 2013). Moreover, the analysis suggests that identity work for MCs encompasses a broad range of considerations beyond the acquisition of military cultural competence. These include, but are not limited to: managing internal dialogues between salient priestly and military identities (which may vary depending on one’s path into the role of MC); negotiating appropriate degrees of closeness and distance; navigating between divergent cultural perspectives; managing potential conflicts of interest; organizing working time; addressing reactions to military identity markers within parish settings; coping with experiences of loneliness or a sense of not fully belonging to any single context; and addressing gender-related dynamics.

Regardless of whether an individual assumes the position of MC with a distinct military identity—that is, already holding dual identities as soldier/officer and priest—or enters the role without a military background or identity, the position of MC is inherently that of a hybrid professional role. It is constituted through intersecting categories and encompasses both explicitly clerical and military elements. This hybridity is not formally regulated in a clear or systematic manner; rather, it emerges through what may be described as an implicit work contract (Watson, 2017, p. 270).

### The implicit work contract and hybridity in the complex, boundary-spanning profession of military chaplaincy

Contemporary sociology of work has increasingly emphasized the importance of identity, belonging, and normative regulation, particularly in relation to complex and boundary-crossing professional roles. Sennett (1998, p. 38, 170-171, 137-138) argues that work is not merely an economic activity but a central source of identity, meaning, and moral orientation. Hynes et al. (2025) have specifically highlighted the lack of moral orientation in a military context, which may occur when chaplains are absent to support commanders in moral orientation and ethical decision-making.

Similarly, Watson (2017, p. 290-293) highlights how professional identities are constructed through the interplay between individual narratives and organizational norms, often negotiated through what he terms a negotiated order, where the implicit work contract plays a pivotal role.

The implicit work contract refers to the unwritten expectations, norms, and cultural codes that shape both work practices and professional identity, yet are rarely formalized in organizational policy (Watson, 2017, p. 290). For instance, MCs may be issued an assault rifle and a handgun during deployment, but nowhere does it state that they are required to kill. Nevertheless, they are expected to do so if necessary. This can have profound consequences for their vows of ordination and identity work, laden with inner conflict, cognitive dissonance, and even moral injury. While the official duties of MCs are explicitly defined (2024a), there are also implicit expectations not written down in an employment contract that they are nonetheless expected to fulfill. In the case of MCs—priests serving within the armed forces—a particularly complex dynamic emerges in which two institutional logics intersect: the ecclesiastical and the military. Neither the Church of Sweden nor the Swedish Armed Forces explicitly regulates how military culture, the presence of weapons, or hierarchical structures impact the priesthood. Nevertheless, these elements become highly relevant in professional practice.

It is through the implicit work contract that such norms and expectations are internalized. Military culture promotes ideals such as discipline, loyalty, strength, hierarchy, and the use of violence—values that interact with clerical identity to form a hybrid professional identity as an MC. In this context, the individual must navigate between potentially conflicting ethical frameworks and normative systems that may give rise to *cognitive dissonance* (Festinger, 1957). This process entails continuous identity dialogue and negotiation (Hermans, 1996, 2001, 2002), in which it is not always clear which logic—that of the Church, the military, or a hybrid of the two—should take precedence.

Importantly, the relationship between military and ecclesiastical logics is not solely characterized by conflict. There are also significant areas of convergence. Ideals such as serving a higher purpose, submission to authority, willingness to sacrifice, discipline, and loyalty are core values shared by both institutions (Grimell, 2021b). This shared value base may facilitate the integration of clergy into military contexts and provide existential meaning and motivational grounding for chaplains. However, such overlaps also risk blurring critical lines of demarcation—boundaries that separate priestly vows and the priesthood from service within a state institution authorized to employ violence (Grimell, 2021a, 2021b).

Here, the concept of institutional complexity becomes salient, referring to the coexistence of multiple institutional logics within a single organizational setting (Greenwood et al., 2011). When these logics are not fully aligned—and instead impose competing normative demands—they may produce professional role stress (Kahn et al., 1964; Peterson et al., 1995). MCs may be expected, in some situations, to prioritize military needs (e.g., unit cohesion, operational effectiveness, support for combat operations, or even the use of lethal force in self-defense), and in others, to uphold ecclesiastical norms (e.g., the integrity of soul care, the practice of forgiveness, the embrace of the weak and broken, and a commitment to nonviolence). Operating within this tension requires constant identity work and boundary negotiation (Watson, 2017, p. 207, 290, 303–305), particularly in an organizational environment as culturally pervasive and normatively intensive as the military (Hall, 2012a,b; Huntington, 1957, p. 11–12, 14, 16; Thornborrow and Brown, 2009; Woodward and Jenkings, 2011).

Thus, while shared values between the Church and the Armed Forces can provide a foundation for integration, it remains crucial to analytically identify and maintain the boundaries that safeguard professional integrity, ensure organizational sustainability, and minimize role stress in these hybrid roles. Understanding how hybrid professional identities are shaped—and the pressures they entail—offers critical insight into how individuals manage, reproduce, or transform cultural norms in complex organizational environments.

While implicit employment contracts afford considerable flexibility to both individuals and organizations—a feature that can be particularly advantageous for MCs, the Church, and the Armed Forces—they also leave significant scope for interpretation in various respects. Establishing clearer and more explicit contractual frameworks may benefit both commissioning bodies and the identity work of MCs, particularly in relation to working hours, role expectations, task delineation, boundary management, and fostering a more structured dialogue between the Church and the Armed Forces. Such measures could also support the development of constructive solutions aimed at increasing the presence of MCs in military contexts where their engagement is sought. Furthermore, the wider dissemination of knowledge regarding the role of MCs within both ecclesiastical and military settings could help clarify critical aspects that may mitigate or eliminate potential areas of tension in identity work.

### Limitations and future research

This study employed a qualitative method, which means that the findings cannot be generalized to the entire population of MCs in Sweden. However, it is reasonable to assume that MCs who were not included in this study may also recognize themselves in the experiences reported by the participants and in the patterns that have emerged.

The findings are also likely to resonate, to varying degrees, with MCs in neighboring Nordic and Scandinavian countries where armed chaplains are likewise present, or at least where the possibility of being armed exists. Although MCs in other unarmed chaplaincy services may experience different degrees of militarization upon entering their positions, the likelihood of conflict and potential cognitive dissonance is lower due to the absence of weapons. In both the Nordic and Scandinavian cases, as well as in relation to unarmed chaplaincy services more broadly, these remain ultimately empirical research questions that must be investigated before conclusions can be drawn about their applicability to these contexts.

One limitation of using a qualitative questionnaire with open-ended questions is that the researcher was unable to pose follow-up questions in cases where responses prompted further curiosity or inquiry. Consequently, certain responses have been interpreted with caution in order to avoid overinterpretation.

Future research on identity work would benefit from more in-depth qualitative approaches, including both one-off interviews and longitudinal studies that follow groups of MCs—with and without military backgrounds—as they enter the role and as their identity work evolves over time. Of particular interest is to investigate, through an interview design, how MCs employ coping strategies to resolve or fail to resolve identity conflicts, cognitive dissonance, and the impact of these processes on their social, personal, and professional lives. In addition, there is the question of whether gender differences exist between men and women, for example due to experiences of a male dominated military culture and the challenges of an identity as a woman, which the current design has not allowed to be explored in a more systematic manner.

## Data Availability

The datasets presented in this study can be found in online repositories. The names of the repository/repositories and accession number(s) can be found in the article/supplementary material.
